# Specific exposure of ICU staff to SARS-CoV-2 seropositivity: a wide seroprevalence study in a French city-center hospital

**DOI:** 10.1186/s13613-021-00868-8

**Published:** 2021-05-13

**Authors:** Emmanuel Vivier, Caroline Pariset, Stephane Rio, Sophie Armand, Fanny Doroszewski, Delphine Richard, Marc Chardon, Georges Romero, Pierre Metral, Matthieu Pecquet, Adrien Didelot

**Affiliations:** 1 Medecine Intensive Reanimation, Centre Hospitalier Saint Joseph Saint Luc, 20 Quai Claude Bernard, 69007 Lyon, France; 2 Maladies Infectieuses, Centre Hospitalier Saint Joseph Saint Luc, Lyon, France; 3 Unite de Recherche Clinique, Centre Hospitalier Saint Joseph Saint Luc, Lyon, France; 4 Medecine du travail, Centre Hospitalier Saint Joseph Saint Luc, Lyon, France; 5 Gestion de La Qualite Et du Risque, Centre Hospitalier Saint Joseph Saint Luc, Lyon, France; 6 Departement D’Information Medicale, Centre Hospitalier Saint Joseph Saint Luc, Lyon, France; 7 Laboratoire de Biologie Medicale, Centre Hospitalier Saint Joseph Saint Luc, Lyon, France; 8 Service de neurologie, Centre Hospitalier Saint Joseph Saint Luc, Lyon, France

**Keywords:** COVID-19, SARS-CoV-2 seroepidemiologic studies, Health personnel

## Abstract

**Background:**

Most hospital organizations have had to face the burden of managing the ongoing COVID-19 outbreak. One of the challenges in overcoming the influx of COVID-19 patients is controlling patient-to-staff transmission. Measuring the specific extent of ICU caregiver exposure to the virus and identifying the associated risk factors are, therefore, critical issues. We prospectively studied SARS-CoV-2 seroprevalence in the staff of a hospital in Lyon, France, several weeks after a first epidemic wave. Risk factors for the presence of SARS-CoV-2 antibodies were identified using a questionnaire survey.

**Results:**

The overall seroprevalence was 9% (87/971 subjects). Greater exposure was associated with higher seroprevalence, with a rate of 3.2% [95% CI 1.1–5.2%] among non-healthcare staff, 11.3% [8.9–13.7%] among all healthcare staff, and 16.3% [12.3–20.2%] among healthcare staff in COVID-19 units. The seroprevalence was dramatically lower (3.7% [1.0–6.7%]) in the COVID-19 ICU. Risk factors for seropositivity were contact with a COVID-19-confirmed household (odds ratio (OR), 3.7 [1.8–7.4]), working in a COVID-19 unit (OR, 3.5 [2.2–5.7], and contact with a confirmed COVID-19 coworker (OR, 1.9 [1.2–3.1]). Conversely, working in the COVID-19-ICU was negatively associated with seropositivity (OR, 0.33 [0.15–0.73]).

**Conclusions:**

In this hospital, SARS-CoV-2 seroprevalence was higher among staff than in the general population. Seropositivity rates were particularly high for staff in contact with COVID-19 patients, especially those in the emergency department and in the COVID-19 unit, but were much lower in ICU staff.

*Clinical trial registration* NCT04422977

**Supplementary Information:**

The online version contains supplementary material available at 10.1186/s13613-021-00868-8.

## Background

Since its outbreak in January 2019 in Wuhan, China, the coronavirus disease 2019 (COVID-19) pandemic has burdened healthcare systems worldwide [1–3]. As suspected and recently identified, one of the main difficulties in managing the outbreak has been the specific exposure of hospital staff [4–7]. Evidence and an understanding of the risk factors for staff infection remain lacking, however. Hospitals concentrate many infected patients and may be significant spreaders of the disease. Intra-hospital clusters among staff may emerge and induce secondary nosocomial infections. The effectiveness of personal protective measures and equipment for caregivers has been well demonstrated, but its use remains imperfect for several reasons: insufficient training, errors of use, and caregivers fatigue [8, 9]. The spread of COVID-19 has been facilitated by the frequency of mildly symptomatic and asymptomatic forms, and by the length of the incubation period of the virus [10]. Contact tracing and screening and the tracking of intra-hospital epidemic outbreaks are, therefore, very difficult [11, 12]. The unusual and rapid influx of patients has required hospitals to reorganize, with the accelerated training of healthcare teams further increasing the risk of an imperfect implementation of protective measures.

We report a large prospective study of severe acute respiratory syndrome coronavirus 2 (SARS-COV-2) seroprevalence among the staff of a city-center hospital in Lyon, France, where there was an influx of COVID-19 patients in March–April 2020. The aims of the study were to determine the overall SARS-COV-2 seroprevalence rate in the hospital staff, and to identify and quantify risk factors for symptomatic and asymptomatic staff infection. The main research questions were (i) whether seroprevalence was higher in healthcare personnel than in non-healthcare staff, (ii) whether working in units managing COVID-19 patients increased the risk of infection and (iii) whether a specific exposure was associated with working in intensive care unit.

## Methods

### Hospital description and epidemic background

**Fig. 1:**
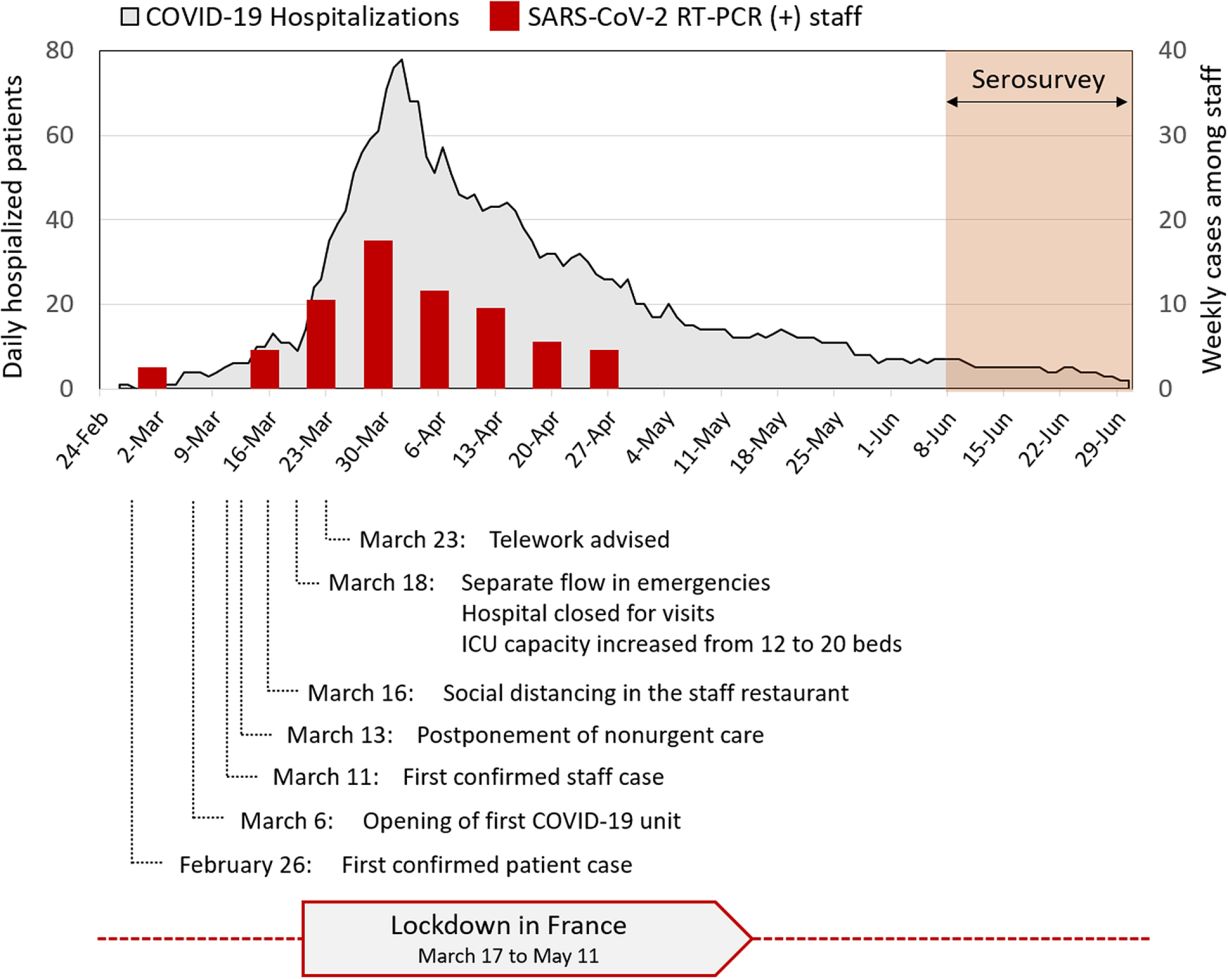
Timeline of daily COVID-19 hospitalizations and weekly numbers of staff members diagnosed with SARS-CoV-2 by RT-PCR in the studied hospital. The outbreak of the epidemic is highlighted by the daily evolution of COVID-19 hospitalizations (gray curve) and the weekly number of SARS-CoV-2-positive RT-PCR tests among staff (red bars). Key dates are shown at the bottom of the graph. The survey period ran from June 8 to June 30, 2020, 1 month after the end of the lockdown in France. COVID-19: coronavirus disease 2019; SARS-Cov-2: severe acute respiratory syndrome coronavirus 2; RT-PCR: reverse transcriptase polymerase chain reaction; ICU: intensive care unit

Saint Joseph Saint Luc Hospital is a tertiary city-center hospital located in Lyon, in the Auvergne-Rhône-Alpes region in France, where the first wave of the COVID-19 pandemic occurred in March and April 2020. The hospital was strongly involved in tackling the epidemic, mainly through its emergency department and intensive care unit (Fig. 1). The first case was detected on February 26th, and 250 patients with confirmed COVID-19 were eventually hospitalized until June, including 11% in the intensive care unit. The rapid influx of a large number of COVID 19 patients relative to the hospital’s capacity (320 beds) meant that an equally rapid reorganization of the hospital’s treatment units was required. Elective surgery was stopped and specific COVID units were opened to screen patients with suspected COVID-19 (and await CT and/or RT-PCR results) and treat patients with confirmed COVID-19. The capacity of the intensive care unit was increased by 40%. Work meetings were limited and when possible, staff were placed on leave or worked remotely. Family visits were banned. The staff restaurant remained open, but strict social distancing measures were enforced. Each confirmed case among staff was investigated to limit further intra-hospital infections by contact tracing and screening of symptomatic suspects.

### Study design

After the first European wave of the epidemic had passed, SARS-CoV-2 antibody testing and a dedicated survey questionnaire were offered to all persons who had worked at the Saint Joseph Saint Luc hospital in Lyon, Auvergne-Rhône-Alpes, France, from March 1 to April 30 2020. This included all clinical and nonclinical employees, the staff of the subcontracted cleaning company, medical interns and nursing students. Testing was delayed by 6 weeks after the end of the first wave to maximize the sensitivity of the antibody test. Enrollment was free, anonymous and voluntary. The study was registered at ClinicalTrials.gov (NCT04422977) and was approved by the local ethics committee (*Comité de Protection des Personnes SUD-EST* II, Bron, France). Written informed consent was obtained from all participants. The inclusion period ran from June 8 to June 30, 2020, 1 month after the end of the lockdown in France.

### Survey

Participants were asked to complete a dedicated questionnaire (see Questionnaire in Additional file 1). The following parameters were recorded: age, gender, presence of symptoms during the outbreak, and prior COVID-19 RT-PCR results. To determine risk factors for COVID-19 infection, participants were asked to state their occupation, the main locations of their work in the hospital, whether they were in direct contact with patients (healthcare workers) or not (non-healthcare workers) and whether they worked day or night shifts. They were also asked to indicate whether they had been in contact with a RT-PCR confirmed COVID-19 household, colleague or patient. Finally, participants were asked about their use of personal protective equipment, social distancing, where they worked in the hospital, and any mandated work time reduction or telework.

### Serological analysis

The samples were collected in lithium heparin gel separator tubes, centrifuged and kept at − 20 °C until analysis. All tests were carried out after calibrating the analyzer. Before performing the test, the samples were thawed and re-centrifuged. The SARS-Cov-2 serology tests were performed using electrochemiluminescence immunoassays (Elecsys Anti-SARS-CoV-2, C6000, E601 analyzer Roche Diagnostics). In these tests, a recombinant nucleoprotein is used to detect total anti-SARS-CoV2-2 immunoglobulins against nucleopcapsid antigen (IgA, IgM and IgG), a technique whose sensitivity and specificity 14 days after a positive SARS-Cov-2 RT-PCR test have been reported as > 99% [13–15]. Participants were considered seropositive for SARS-CoV-2 if their test results were above the manufacture-specified threshold.

### Statistical analysis

As the population seroprevalence of SARS-CoV-2 in Auvergne-Rhône-Alpes was unknown at the time of designing the study, the necessary sample size was calculated considering hypothetical seropositivity rates of 15% in unexposed patients and 30% in exposed patients. The estimated sample size required to detect a statistically significant difference between exposure and non-exposure at an α level of 0.05 and a power (1 − β) of 0.9 was 100 participants per group. The expected participation rate was 70% of staff, i.e., 900 persons.

Overall and group-specific seroprevalences were calculated as the ratios of subjects tested with positive serology tests divided by the total number of subjects in each group, expressed as absolute and relative frequencies. Ninety-five percent confidence intervals were estimated using the asymptotic approximation. Age was expressed as median [IQP]. In univariate analysis, proportions were compared using χ^2^ tests or Fisher exact tests (depending on sample size) and ages were compared using Mann–Whitney U tests. Reported symptoms and exposure factors associated with positive serology tests were then successively assessed by means of multivariate logistic regression analyses. The maximal model included all exposures associated at *P* < 0.15 with a positive serology test. The final model included all variables significantly associated (*P* < 0.05, two tailed) with a positive serology test. All statistical analyses were performed with SPSS version 20 (IBM, Chicago, IL).

## Results

Among the 1299 staff who worked in the hospital during the first wave of the COVID-19 epidemic, 971 (75%) completed the questionnaire survey and were tested for anti-SARS-CoV-2 Ig antibodies. (Additional file 2: Figure S1). These respondents were representative of all occupations and areas in the hospital (Additional file 3: Table S1 and S2). They included 155 physicians, 274 nurses, 111 nursing assistants, 282 support and administrative staff, and 149 others. Eighty-seven subjects had positive SARS-CoV-2 antibody tests, such that the overall seroprevalence in the study group was 9% [8.8–9.2%]. There was no significant association between SARS-CoV-2 seropositivity and sex, (male sex, 20/87 [23.0%] vs 213/884 [24.1%], *P* = 0.87) but seropositive staff were slightly younger on average (median age, 37 [28–47] vs 39 [31–51] years, *P* = 0.02).

### Symptoms

Forty-two percent of subjects (410/971) reported at least one prior symptom but this was not a highly sensitive ([76/87] 87%) or specific ([550/884] 62%) sign. Of the 87 members of staff with positive SARS-CoV-2 antibody tests, 11 (12.6%) reported no prior symptoms. Overall, the positive predictive value of declaring one or more prior symptoms was very low (76/410 [18%]). However, anosmia (OR = 55.3) and fever (OR = 3.5) were strongly associated with subsequent seropositivity (Additional file 4: Table S3). Fifty of the 87 subjects with positive SARS-CoV-2 antibody tests had previously had a RT-PCR test for SARS-CoV-2, which was positive in 41/50 cases (84%). One subject who had had a positive RT-PCR test had a negative antibody test.

### Exposure factors associated with SARS-CoV-2 seropositivity

**Fig. 2:**
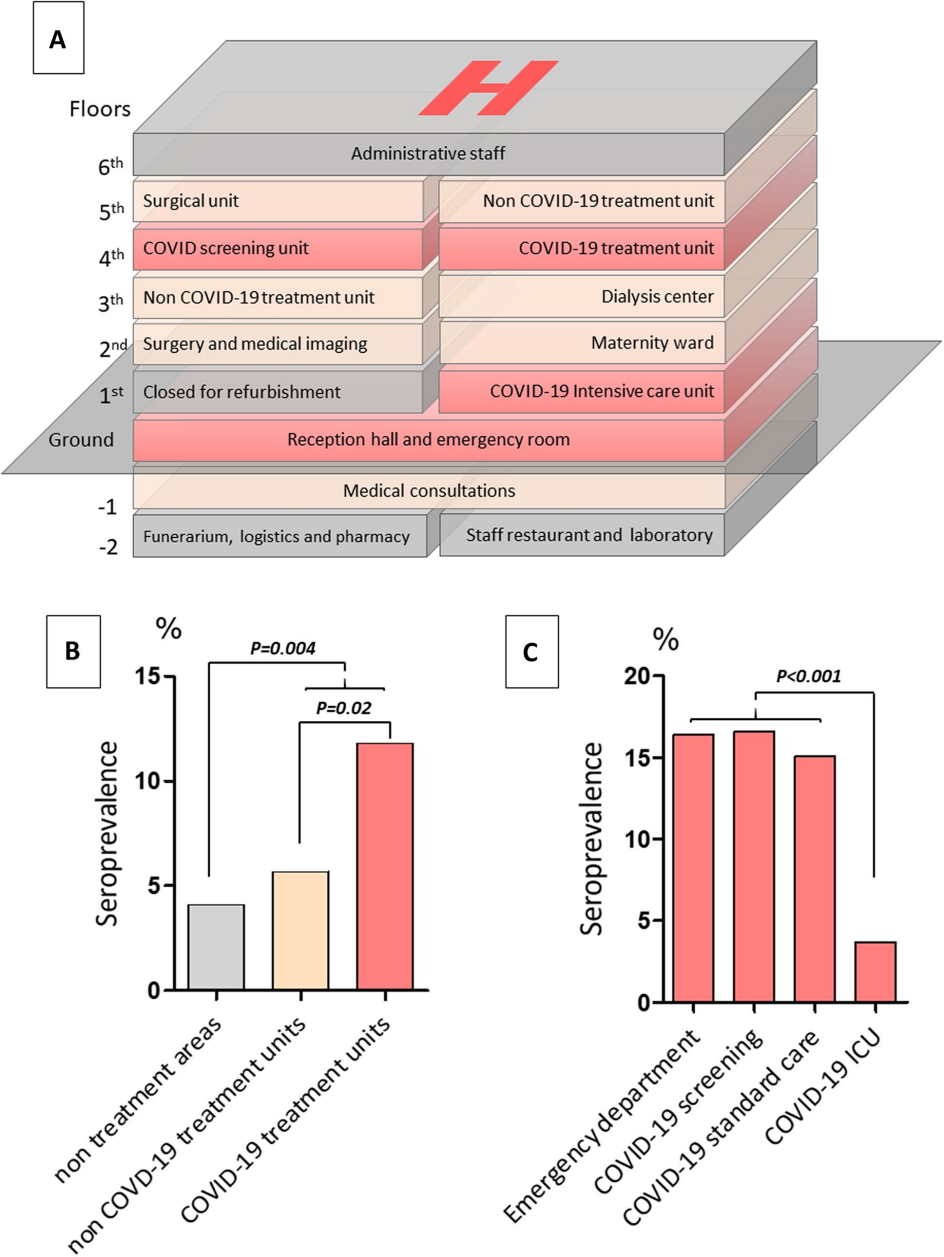
Risks of exposure to SARS-CoV-2 infection in different areas of the hospital. **a** Schematic illustration of the hospital's single-block architecture with administrative and support areas in gray, non-COVID treatment units in pink and COVID-19 treatment units in red. Surgical units include: plastic and reconstructive surgery, urology, vascular surgery, orthopedics, gynecology and digestive surgery (5th Floor). Non-COVID-19 treatment units include: nephrology, endocrinology, rheumatology, gastroenterology, neurology, infectious disease, internal medicine, cardiology (3rd Floor) and cardiologic intensive care unit (5th Floor). **b**, **c** Staff SARS-CoV-2 seroprevalence (**b**) in the different work areas (non-treatment areas, non-COVID-19 treatment areas, COVID-19 treatment areas) and **c** in the different COVID-19 units. COVID-19: coronavirus disease 2019; SARS-Cov-2: severe acute respiratory syndrome coronavirus 2; ICU: intensive care unit

Being involved in clinical care was significantly associated with SARS-CoV-2 seropositivity (78/689 positive antibody tests [11.3%] among healthcare personnel vs. 9/282 [3.2%] in non-healthcare personnel; *P* < 0.001) (Table 1). In univariate analysis, the seropositivity rate was particularly high among nurses (38/274 [13.9%], *P* = 0.001) and was also significantly higher in staff who reported working in COVID-19 units than in those who reported working in non-COVID-19 units (68/577 [11.8%] vs 10/174 [5.7%], *P* = 0.02) (Table 2). The prevalence of seropositivity was especially high in emergency department staff, the COVID-19 screening unit and the COVID-19 treatment unit, but much lower in those working in the COVID-19-ICU, with respective rates of 26/159 (16.4%), 35/211 (16.6%), 28/185 (15.1%) and 7/191 (3.7%) (Fig. 2).

**Table 1:** Proportion of SARS-CoV-2 seropositive staff by occupation,

Function	Workers per occupation	SARS-CoV-2 seropositive staff in the category (%)	SARS-CoV-2 seropositive staff out of the category (%)	P*
All staff	971	9	–	–
Healthcare staff	689	11.3	3.2	< 0.001
Physicians	155	12	8.2	0.06
Nurses	274	13.9	7	0.001
Nursing assistants	111	12.6	8.5	0.15
Midwives	27	3.7	9.1	0.33
Radiology technicians	23	4.3	9.1	0.71
Cleaning staff	39	7.7	9	> 0.99
Porters	11	9.1	9	> 0.99
Others	49	0	9.4	0.02
Non-healthcare staff	282	3.2	11.3	< 0.001
Management staff	52	0	9.5	0.01
Nurse managers	37	0	9.3	0.07
Medical secretaries	70	5.7	9.2	0.39
Laboratory and sterilization technicians	43	4.7	9.2	0.42
Caterers	10	10	8.9	0.61
Others	70	2.9	9.4	0.79

SARS-CoV-2: severe acute respiratory syndrome coronavirus

*Proportions of seropositive staff in each category are compared to the proportions of seropositive staff in the other categories with χ^2^ test or Fischer exact test

**Table 2:** Exposure factors associated with SARS-CoV-2 seropositivity in hospital staff. Univariate analysis

Exposure factor	Proportion of SARS-CoV-2 seropositive staff (%)		*P*
	Exposure present	Exposure absent	
Contacts			
COVID-19 + coworker	33/195 (16.9)	54/776 (7.0)	0.001
COVID-19 + household	14/54 (25.9)	73/917 (8.0)	< 0.001
COVID-19 + patient	29/162 (17.9)	58/809 (7.2)	< 0.001
Work areas			
Any care unit	78/752 (10.4)	9/219 (4.1)	0.004
Emergency department	26/159 (16.4)	61/812 (7.5)	0.001
COVID-19-free medical unit	12/160 (7.5)	75/811 (9.2)	0.48
COVID-19-free surgical unit	11/119 (9.2)	76/852 (8.9)	0.91
Operating room	3/114 (2.6)	84/857 (9.8)	0.01
COVID-19 screening unit	35/211 (16.6)	52/760 (6.8)	< 0.001
COVID-19 standard care unit	28/185 (15.1)	59/786 (7.5)	0.001
COVID-19 intensive care unit	7/191 (3.7)	80/780 (10.3)	0.004
Transmission prevention measures			
Optimal use of personal protective equipment	52/575 (9)	35/396 (8.8)	0.91
Meals in the staff restaurant	22/340 (6.5)	65/631 (10.4)	0.05
Meals in a break room of a COVID-19 unit	45/259 (17.4)	42/712 (5.9)	< 0.001
Working time reduction ≥ 50%	5/103 (4.9)	82/868 (9.4)	0.12
Telework	7/194 (3.6)	80/777 (10.3)	0.004

COVID-19: coronavirus disease 2019; SARS-Cov-2: severe acute respiratory syndrome coronavirus

Reporting a personal contact with a confirmed COVID-19 household, a confirmed COVID-19 coworker or a confirmed COVID-19 patient were all significantly associated with higher seropositivity rates (33/195 [16.9%], 14/54 [25.9%] and 29/162 [17.9%], respectively). On the other hand, working in the operating room, eating meals break in the staff restaurant, and telework during the lockdown were associated with lower incidences of positive antibody tests (3/114 [2.6%], 22/340 [6.5%] and 7/194 [3.6%], respectively). Night work and the ability to maintain social distancing at work were not significantly associated with the seropositivity rate (data not shown). Table 2 lists all the univariate associations between exposure factors and antibody positivity. In multivariate logistic regression analysis, the three exposure factors positively associated with a higher seropositivity rate were contact with a confirmed COVID-19 household, contact with a confirmed COVID-19 coworker and working in a non-intensive care COVID-19 unit, with estimated odds ratios of 3.7 [95% CI 1.8–7.4], 1.9 [95% CI 1.2–3.1] and 3.5 [95% CI 2.2–5.7], respectively (Table 3). On the other hand, working in the COVID-19 ICU was associated with a lower rate of positive antibody tests (OR = 0.33, [95% CI 0.15–0.73]).

**Table 3:** Exposure factors associated with SARS-CoV-2 seropositivity in hospital staff

Exposure factors	Odds ratio (95% CI)	*P*
Contact with COVID-19 + coworker	1.9 (1.16–3.12)	0.01
Contact with COVID-19 + household	3.67 (1.83–7.38)	< 0.001
Worked in standard care COVID-19 unit	3.54 (2.2–5.7)	< 0.001
Worked in COVID-19 ICU	0.33 (0.15–0.73)	0.007

Results of multivariate analysis by stepwise backward logistic regression

COVID-19: coronavirus infectious disease 2019; ICU: intensive care unit

## Discussion

This cross-sectional seroprevalence study confirms that hospital workers may have a higher risk of SARS-CoV-2 infection. The risk seems to be particularly high for staff in direct contact with COVID-19 patients, the groups with the highest seropositivity rates in this study being emergency department and COVID-19 unit workers. In contrast, ICU staff had relatively lower seropositivity rates.

### Global seroprevalence

The overall SARS-CoV-2 seroprevalence rate among hospital staff (9%) was twice the estimated community seroprevalence in the hospital catchment area during the screening period (3–5%) [16, 17]. The prevalence of infections among hospital staff across Europe was characterized by a great diversity during the first phase, ranging from 2 to 30% according to countries and area [5, 18–20]. This high variability is related not only to local prevalence of community infection but also to specific hospital measures to contain the infection.

This result is significant, because only 41/87 subjects had previously been diagnosed by RT-PCR. While RT-PCR remains essential for contact tracing, this technique is limited by its low sensitivity and the fact that the virus may no longer be present at the time of screening [21]. The positive predictive value of a symptom declaration for positive serology was very weak in our study, this uncommon result could be explained by technical (weak sensibility of the electrochemiluminescence test) or methodological biases (suggestive questionnaire, nonspecific symptoms, long study period, sero-reversion before the survey). Furthermore, the rate of asymptomatic seropositive workers was only 13%, which contrasts with other series (20–30%) [20, 22]. This low rate could also be explained by overdeclaration of symptoms, low sensitivity of the test, possible sero-reversion and a global low seroprevalence in our cohort.

### Exposure risk factors

In keeping with the results of a recent hospital-wide survey in Belgium, we found that the primary risk factor for SARS-CoV-2 seropositivity was contact with a confirmed COVID-19 household [19]. However, this factor must be interpreted with caution, as the delay between contact and the seroprevalence study makes it impossible to determine whether the contact with confirmed COVID-19 was indeed the initial source of the infection or was secondarily infected by the member of staff. Our study also highlights the risk of intra-hospital spread, contact with a COVID-19-positive coworker being associated with a twofold increase in the risk of SARS-CoV-2 seropositivity. Working in units where COVID-19 patients were treated was a strong risk factor for subsequent SARS-CoV-2 seropositivity. This may be due in part to the shortage of personal protective equipment at the start of the epidemic, as reported in other series [20, 23]. Another special difficulty was to apply social distancing measures in a hospital environment, especially during the staff meals. Indeed, eating in a break room of a COVID care unit was strongly associated with a higher seroprevalence, in univariate analysis. Saint Joseph Saint Luc is a small single-block city-center hospital with small and tightly packed treatment areas. By contrast, having lunch in the staff restaurant seems to have been a protective factor. The restaurant is more spacious and ventilated and the social distancing seems to have been better defined and respected there.

A similar seroprevalence study conducted in a Spanish hospital facing a higher local epidemic wave within the same period reveals some concordant conclusions: higher infection rate among caregivers vs. non-caregivers [20]. But differently from us, the authors showed the lack of PPE and a previous contact with COVID-19 patients were independent factors that were associated with SARS-CoV-2 infection. The seroprevalence was the highest among porters.

We found that staff treating COVID-19 patients in the ICU had a much lower than average SARS-CoV-2 seropositivity rate. This result is in line with a similar previous study [22]. It is of particular clinical significance as it concerns both regular ICU staff and (about 90) additional crisis support personnel. This relatively low seroprevalence may stem from the particular focus on intensive care at the start of the epidemic, with less shortage in protective personal equipment and high standards of infection control. The closed circuit ventilation of intubated patients may also have limited the spread of the virus and the risk of aerosolization with non-invasive techniques seems to be limited [24, 25]. The staff/patient ratio is higher in intensive care than in standard care units with lower patient turnover, which may have reduced individual risks of exposure [26]. Furthermore, patients are often admitted to intensive care 5–8 days after first presenting symptoms and may, therefore, have a lower viral load during their ICU stay [27].

Interestingly, ICU workers had no less household contact but significantly less COVID-19 coworker contact than others hospital workers (5.1% vs. 5.7%, *P* = 0.76 and 20.9% vs 30.1%, *P* = 0.01). These data highlight the responsibility of staff clusters in the intra-hospital spread of SARS-Cov-2.

### Strengths and limitations

The strengths of the study are the large number of staff included throughout the hospital (including both healthcare and non-healthcare workers), the short data collection time and the optimal delay between the peak of the epidemic and the survey. Recent data show that seroconversion can be delayed by up to 3 weeks after infection with SARS-CoV-2, especially in cases of mildly symptomatic disease [28]. This could explain the discrepancies between the results of our study and those of Steensels et al. [19].

The limitations of the study are first, its single center design, which means that any generalization of the results should be made with caution. Second, since some staff members chose not to participate in the study, selection bias cannot be excluded. Third, to respect patient confidentiality, the questionnaires were self-administered and anonymous, leading to possible errors or misunderstandings. Fourth, we used an automated electrochemiluminescence immunoassay to detect total anti-SARS-CoV-2 antibodies and not a differential detection of IgM and igG with ELISA (enzyme linked immunosorbent assay); however, recent data suggest a good agreement between the two methods [14]. Fifth, given the three-month delay between the first case of COVID-19 in a healthcare worker and the survey, some degree of sero-reversion may have contributed to underestimating global seroprevalence. Recent data suggest that antibody responses could decrease rapidly (especially in moderate forms) [29, 30].

## Conclusion

In this study, hospital staff treating COVID-19 patients were at a high risk of intra-hospital infection. This suggests that prevention measures should be improved and infection clusters tracked carefully in COVID-19 units. Our results indicate that the risk of exposure seems to be unevenly distributed throughout hospitals, seropositivity rates being higher than average in the emergency department and (standard care) COVID-19 unit where patient turnover was high, and this is where prevention efforts should be focused in managing the epidemic. The relatively low seropositivity rate observed in COVID-19 intensive care staff suggests that intra-hospital patient-to-staff transmission is limited in this area.

## Supplementary Information

**Additional file 1.** Questionnnaire for participants.

**Additional file 2: Figure S1.** Enrollment of study participants.

**Additional file 3: Table S1.** Stratification of the sample by work area. **Table S2.** Participation rates according to healthcare workers category.

**Additional file 4: Table S3.** Symptoms associated with SARS-CoV-2 seropositivity in hospital staff.

## Data Availability

The datasets used and/or analyzed during the current study are available from the corresponding author on reasonable request.
