# Supplementary material for: Specific exposure of ICU staff to SARS-CoV-2 seropositivity: a wide seroprevalence study in a French city-center hospital
Source: Ann Intensive Care. 2021 May 13;11:75. doi: 10.1186/s13613-021-00868-8 (PMC8118099; doi:10.1186/s13613-021-00868-8)
Supplement: Supplementary file 1 — Additional file 1. Questionnnaire for participants. [file 13613_2021_868_MOESM1_ESM.docx]

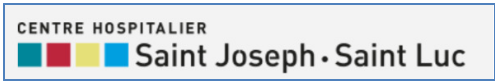
SEROPHUGAC study Subject №/Label

Questionnaire for participants

Age: ____ years Sex:  Male  Female

MEDICAL HISTORY

**Have you had any symptoms of possible CORONAVIRUS infection over the past 3 months (between February and early May)?**

 YES  NO

If so, please specify which ones:

 Fever,  Cough,  Aches and pains,  Headache,  Difficulty breathing,  Chest pain,  Extreme tiredness,  Diarrhe,  Loss of taste or smell

**Have you had one (or several) RT-PCR tests for CORONAVIRUS infection?**

 YES

 NO

Date:

If so, was the test positive?

 YES

 NO

**Have you had a CT scan to diagnose CORONAVIRUS infection?**

 YES

 No

If so, was the diagnosis positive?

 YES

 NO

**Have you had an antibody test for CORONAVIRUS infection?**

 YES

 NO

Date:

If so, was the test positive?

 YES

 NO

Where was the sample taken?

**Overall, do you think you have been infected by the virus?**

 YES, with certainty

 Probably not,

 Very probably,  NO, certainly not

1/3

SEROPHUGAC study questionnaire


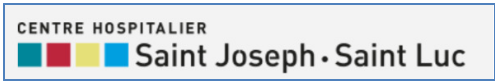
SEROPHUGAC study Subject №/Label

CONTACT SUBJECTS

**Have you been in face-to-face contact, without a mask, for a least 15 minutes with one (or several) individuals with confirmed COVID (positive diagnosis by RT-PCR or CT)?**

 **No**  **Yes**, in which case, please specify (multiple responses possible):

 In a non-professional setting (family members)

 In a professional setting ( colleagues,  patients)

WORKING CONDITIONS

**How would you describe your knowledge of best practices in terms of PPE (personal protective equipment) and infection control measures?**

 Poor  Average  Optimal

**How would you describe your adherence to best practices in terms of PPE (personal protective equipment) and infection control measures?**

 Poor  Average  Optimal

**Where have you (mainly) eaten your meals?**

 In the staff restaurant,

 In a COVID unit break room

 In a non-COVID unit break room

 In an office

 Outside the hospital

**Do you think you were able to keep 1 m apart from other people when you were not wearing a mask?**

 Yes  No, and if not, in what location(s) and situation(s)

**What is your occupation in the hospital?**

|  Physician, |  Medical intern, |  Nurse, |  Nursing assistant, |
| --- | --- | --- | --- |
|  Physiotherapist, |  Porter, |  X-ray tech. |  Nurse manager, |
|  Midwife, |  IT, |  BMET, |  Laundry, |
|  Cleaning, |  Catering, |  Maintenance dept.,  Management, | |
|  Secretary, |  Lab technician, |  Pharmacist, |  Reception, |
| Other: |  |  |  |

2/3

SEROPHUGAC study questionnaire


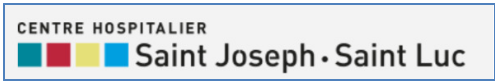
SEROPHUGAC study Subject №/Label

**Did you work in a treatment unit between February and early May?**

 YES  NO,

If so, please specify **which units you mainly worked in** (more than 50% of working time)

|  Emergency |  |  Intensive care |  Operating room | |  Dialysis |
| --- | --- | --- | --- | --- | --- |
|  Maternity |  |  CCU |  Ambulatory |  |  Consultations |
|  3A |  3B |  4A |  4B |  4C |  4D |
|  5A |  5B |  5C |  5D |  |  |

Other:

 I worked in **several units** (multidisciplinary activities)

 I worked in **another treatment center** in this period

**Did you work in a unit treating patients with COVID ?**

 YES  NO,

If so,  ED  Short-stay unit,  Intensive care,  4CD,  Screening unit

Other:

**Did you work**

 During the day  At night  Both in alternation

**Outside mealtimes, did you wear a mask in communal areas (meeting rooms, treatment rooms, medical offices…)?**

 YES  NO,

**Where you able to telework or was your attendance time at the hospital reduced?**

 YES  NO

If so, your attendance time was reduced by:

 10%  25%  50%  >50%

**Thank you for taking the time to complete this questionnaire**

3/3

Questionnaire étude SEROPHUGAC
