# Supplementary material for: Specific exposure of ICU staff to SARS-CoV-2 seropositivity: a wide seroprevalence study in a French city-center hospital
Source: Ann Intensive Care. 2021 May 13;11:75. doi: 10.1186/s13613-021-00868-8 (PMC8118099; doi:10.1186/s13613-021-00868-8)
Supplement: Supplementary file 2 — Additional file 2: Figure S1. Enrollment of study participants. [file 13613_2021_868_MOESM2_ESM.docx]

**Figure S1. Enrollment of study participants**

| **Eligible: 1299** members of staff who worked in Hospital Saint Joseph Saint Luc in March-April 2020 including:   - 1165 hospital employees - 64 employees of the subcontracted cleaning company - 70 nursing students |
| --- |

| **Enrolled: 974** members of staff who completed the survey |
| --- |

| **Excluded: 3** members of staff with no blood sample |
| --- |

| **Included: 971** members of staff who completed the questionnaire and had antibody tests |
| --- |
