# Supplementary material for: Specific exposure of ICU staff to SARS-CoV-2 seropositivity: a wide seroprevalence study in a French city-center hospital
Source: Ann Intensive Care. 2021 May 13;11:75. doi: 10.1186/s13613-021-00868-8 (PMC8118099; doi:10.1186/s13613-021-00868-8)
Supplement: Supplementary file 3 — Additional file 3: Table S1. Stratification of the sample by work area. Table S2. Participation rates according to healthcare workers category. [file 13613_2021_868_MOESM3_ESM.docx]

**Table S1. Stratification of the sample by work area**

| Main work areas | Workers included n (%) |
| --- | --- |
| All staff | 971 |
| Emergency department | 159 (16) |
| Surgical unit | 53 (5) |
| Surgical room | 114 (12) |
| Non COVID-19 treatment unit | 146 (15) |
| COVID-19 intensive care unit | 113 (12) |
| COVID-19 screening unit | 42 (4) |
| COVID-19 treatment unit | 63 (6) |
| Medical consultations | 40 (4) |
| Dialysis center | 52 (5) |
| Medical imaging | 20 (2) |
| Maternity ward | 57 (6) |
| Pharmacy | 16 (2) |
| Laboratory | 35 (4) |
| Staff Restaurant | 10 (1) |
| Administrative offices | 51 (5) |

**Table S2. Participation rates according to health care workers category**

| Workers per occupation | Workers eligible | Workers included | Participation rate (%) |
| --- | --- | --- | --- |
| All staff | 1299 | 971 | 75 |
| Physicians | 182 | 155 | 85 |
| Nurses | 327 | 274 | 84 |
| Nursing assistants | 168 | 111 | 66 |
| Midwives | 37 | 27 | 73 |
| Radiology technicians | 30 | 23 | 77 |
| Cleaning staff | 49 | 39 | 80 |
| Porters | 20 | 11 | 55 |
| Management staff | 75 | 52 | 69 |
| Nurse managers | 49 | 37 | 76 |
| Medical secretaries | 94 | 70 | 74 |
| Laboratory and sterilization technicians | 55 | 43 | 78 |
| Caterers | 15 | 10 | 67 |
| Others | 198 | 119 | 60 |
