# Supplementary material for: Specific exposure of ICU staff to SARS-CoV-2 seropositivity: a wide seroprevalence study in a French city-center hospital
Source: Ann Intensive Care. 2021 May 13;11:75. doi: 10.1186/s13613-021-00868-8 (PMC8118099; doi:10.1186/s13613-021-00868-8)
Supplement: Supplementary file 4 — Additional file 4: Table S3. Symptoms associated with SARS-CoV-2 seropositivity in hospital staff. [file 13613_2021_868_MOESM4_ESM.docx]

**Table S3.** **Symptoms associated with SARS-CoV-2 seropositivity in hospital staff**

| \| Symptoms \| Proportion of SARS-CoV-2 seropositive staff (%) \| \|  \| \| --- \| --- \| --- \| --- \| \|  \| Symptom present \| Symptom absent \| Odds ratio (95% CI)* \| \| Fever \| 51/218 (23.4) \| 36/753 (4.8) \| 3.52 (1.78–6.98) \| \| Cough \| 33/194 (17) \| 54/777 (6.9) \| 0.72 (0.34–1.55) \| \| Myalgia \| 35/156 (22.4) \| 52/815 (6.4) \| 1.23 (0.56–2.72) \| \| Headache \| 44/222 (19.8) \| 43/749 (5.7) \| 1.32 (0.66–2.68) \| \| Dyspnea \| 17/69 (24.6) \| 70/902 (7.8) \| 0.68 (0.24–1.91) \| \| Chest pain \| 16/56 (28.6) \| 71 /915 (7.8) \| 2.1 (0.80–5.56) \| \| Asthenia \| 48/223 (21.5) \| 39/748 (5.2) \| 1.34 (0.66–2.75) \| \| Diarrhea \| 17/78 (21.8) \| 70/893 (7.8) \| 1.3 (0.53–3.18) \| \| Anosmia \| 53/70 (75.7) \| 34/901 (3.8) \| 55.29 (27.15–112.6) \|   *SARS-CoV-2, severe acute respiratory syndrome coronavirus 2*  *** *by multivariate logistic regression analyses* |  |
| --- | --- | --- | --- | --- | --- | --- | --- | --- | --- | --- | --- | --- | --- | --- | --- | --- | --- | --- | --- | --- | --- | --- | --- | --- | --- | --- | --- | --- | --- | --- | --- | --- | --- | --- | --- | --- | --- | --- | --- | --- | --- | --- | --- | --- | --- |
